# The Impact of a 24 Month Housing First Intervention on Participants’ Body Mass Index and Waist Circumference: Results from the At Home / Chez Soi Toronto Site Randomized Controlled Trial

**DOI:** 10.1371/journal.pone.0137069

**Published:** 2015-09-29

**Authors:** Julia Woodhall-Melnik, Vachan Misir, Vered Kaufman-Shriqui, Patricia O’Campo, Vicky Stergiopoulos, Stephen Hwang

**Affiliations:** 1 Centre for Research on Inner City Health, St. Michael’s Hospital, Toronto, Ontario, Canada; 2 Dalla Lana School of Public Health, University of Toronto, Toronto, Ontario, Canada; 3 Division of General Internal Medicine, Department of Medicine, University of Toronto, Toronto, Ontario, Canada; Hunter College, UNITED STATES

## Abstract

Research suggests that individuals experiencing homelessness have high rates of overweight and obesity. Unhealthy weights and homelessness are both associated with increased risk of poor health and mortality. Using longitudinal data from 575 participants at the Toronto site of the At Home/Chez Soi randomized controlled trial, we investigate the impact of receiving a Housing First intervention on the Body Mass Index (BMI) and waist circumference of participants with moderate and high needs for mental health support services. The ANCOVA results indicate that the intervention resulted in no significant change in BMI or waist circumference from baseline to 24 months. The findings suggest a need for a better understanding of factors contributing to overweight, obesity, and high waist circumference in populations who have histories of housing precarity and experience low-income in tandem with other concerns such as mental illness and addictions.

**Trial Registration:**

International Standard Randomized Control Trial Number Register ISRCTN42520374

## Introduction

Housing is an important determinant of health. In general, individuals who experience long-term homelessness also experience worse health outcomes and premature mortality compared to their housed counterparts [1] [2] [3]. Studies conducted in the United States indicate that homeless individuals experience high rates of overweight and obesity. In one study measuring Body Mass Index (BMI) in 5,632 homeless adults in the United States, Koh et al. found that only 1.6% were underweight, whereas 32.3% were classified as obese [4]. Other studies have produced similar results [5] [6] [7]. Tsai and Rosenheck found that 57% of adults experiencing chronic homelessness in the United States are overweight or obese [7].

Research shows that being overweight or obese can have negative health outcomes. Those who experience overweight and obesity are more likely to suffer from chronic conditions such as Type II diabetes, heart disease, certain types of cancers [8] and early mortality [9] [10]. Several factors place homeless individuals at greater risk of overweight and obesity compared to the general population. For example, both poverty and lack of stable housing significantly reduce the ability to maintain a healthy and balanced diet [11], leading to food insecurity. Housing First has been shown to be an effective strategy for assisting homeless populations with mental illness [12] [13] [14] [15]. Housing First involves providing low-barrier, rapid access to housing and mental health support services wherein individuals are given access to independent housing with no sobriety or mental health treatment enrollment or compliance requirements [16]. Research suggests that this programming improves a variety of housing, health, and social outcomes [15] [17] [18]. However, the impact of Housing First interventions on body weight has not been assessed to date.

Housing First interventions are based on the premise that receiving access to stable housing with client-driven supports assists individuals in stabilizing other aspects of their lives such as mental health and substance use [16]. Theoretically, providing persons experiencing homelessness with access to housing and treatment for mental illness could impact weight outcomes. Access to housing offers participants the opportunity to purchase, store, and prepare food of higher nutritional value, which may not necessarily be low in energy. In addition, making the transition to stable housing may modify individual food intake as part of an entire life-style modification., At Home /Chez Soi study participants all had serious mental illness [19] [20], and engagement in treatment could lead to increased use of psychotropic medications which is associated with weight gain and metabolic abnormalities [21] [22]. Additionally, changes in drug and alcohol use can result in weight gain or weight loss. The direction of change in weight status is associated with the type of substance, frequency of use, and gender [23] [24] [25]. The literature to date do not present data on whether housing stability increases or decreases individual energy intake through food consumption.

The At Home / Chez Soi intervention was designed to improve housing and social outcomes and was not designed with the specific intent of normalizing weight. However, the intervention was designed with the intent of stabilizing participants’ health outcomes and lives in general. Therefore, we could hypothesize that weight outcomes could have been stabilized as a result of participating in the intervention. To identify the impact of the Housing First intervention on overweight and obesity, we used the anthropometric measurements of weight, height and waist circumference in a sample of homeless individuals with mental illness from the Toronto site of the At Home/Chez Soi randomized clinical trial. The objective of this paper is to address following research question: Does participation in the treatment arms of the At Home / Chez Soi trial have an impact on BMI or waist circumference 24 months following initial enrollment in the study?

## Methods

**Fig 1:**
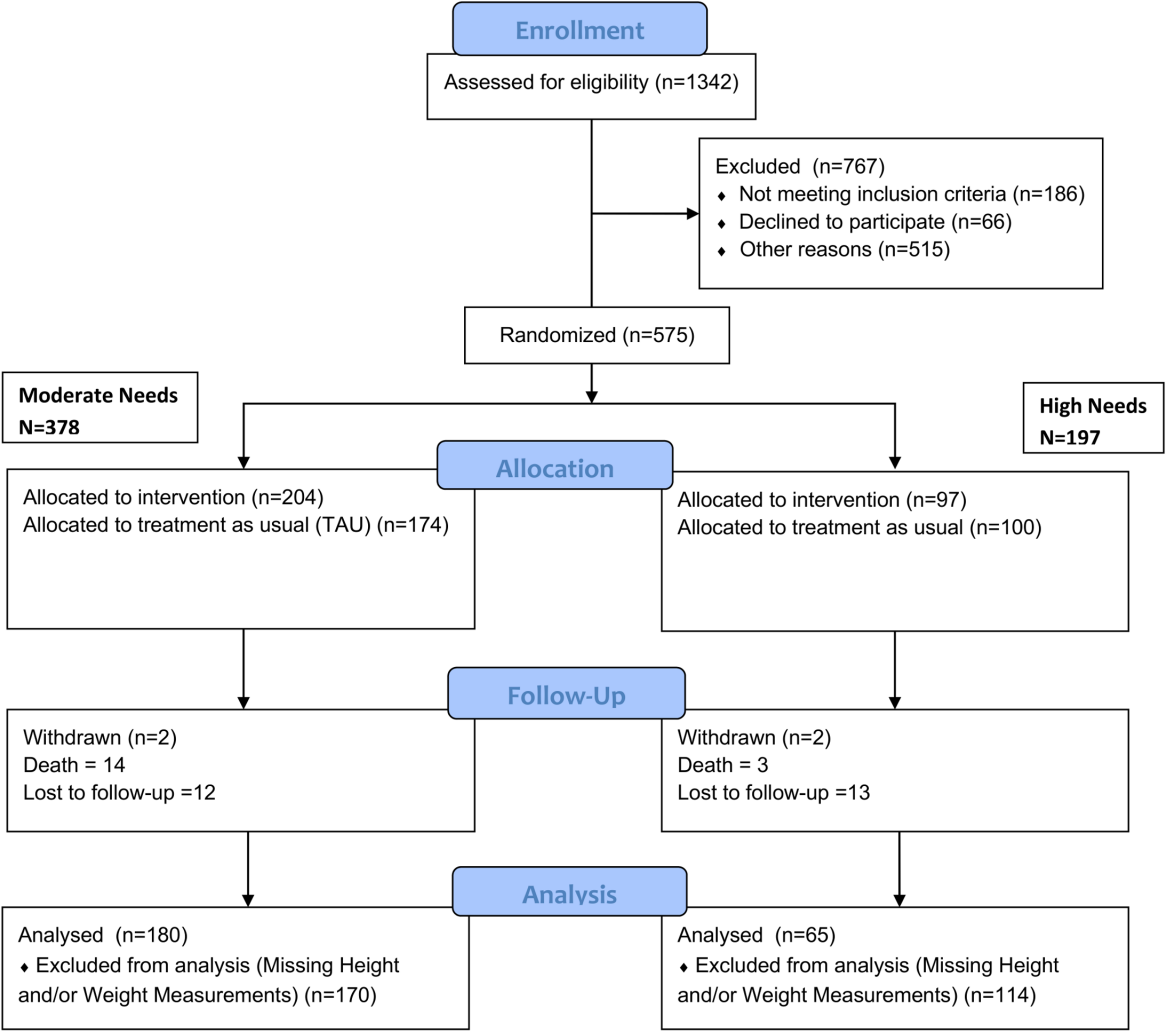
CONSORT Diagram. This figure displays the random assignment of participants to intervention and control groups based on need level and indicates the number of individuals who had data for inclusion in this particular study.

The Toronto At Home/Chez Soi study consists of 575 participants who were placed into moderate and high needs groups based on their scores on the Multnomah Community Ability Scale, the Mini International Neuropsychiatry Interview, the presence of concurrent substance use disorder, acute care utilization and legal involvement [12] [19]. Participants were then randomized to receive Housing First with Assertive Community Treatment (ACT) support vs. Treatment as Usual (TAU) (high needs) or Housing First with Intensive Case Management (ICM) vs. TAU (moderate needs). Adaptive randomization was employed. We used a computer generated algorithm with a central database of participants to complete randomization. The randomization is displayed in Fig 1. Participants in the TAU groups did not receive housing or supports but were provided with lists of resources of community supports which they could choose to access.

Prior to randomization all participants were stratified into need levels based on the extent of their disability and severity of psychiatric problems. To establish the need level of each participant, the community functions, mental disorder diagnoses, comorbid conditions, prior hospitalizations and incarcerations were examined as well as the results from the MINI and Multnomah Community Ability Scale (MCAS). To be considered ‘high needs,’ participants had to have a score of less than 62 and have a MINI diagnosis of a psychotic disorder or bipolar disorder, a prior hospitalization for mental illness at least 2 times in any one year over the last 5 years and either a co-morbid substance use or a recent arrest or incarceration. All other participants who did not meet these criteria were considered ‘moderate needs’. For additional information, see Hwang et al. [19].

Intake coordinators assessed participants for eligibility and then conducted a screening interview prior to placing their information in a centralized database for randomization. To be eligible for participation, respondents were required to be at least 18 years of age, absolutely homeless or precariously housed, have a serious mental disorder with or without a co-occurring substance use problem, and not be presently enrolled in ICM or ACT. Our aim was to recruit 560 participants to detect an effect size of 0.5 for those receiving ICM and ACT. This required that we maintain 63 participants per treatment arm. The recruitment target minimums were set at 100 participants per arm to account for probable attrition.

This study was approved by the Research Ethics Board of St. Michael’s Hospital in Toronto, Ontario (#09–208). Written consent was collected for all study participants. The study was registered with the International Standard Randomized Control Trial Number Register (ISRCTN42520374). The registration is listed at http://www.isrctn.com/ISRCTN42520374. In order to protect participant anonymity, the data used in the following analyses are not publically available. Data access requests can be made by contacting Carol Adair at ceadair@ucalgary.ca.

### Data Collection

The intake period was from October 2009 to June 2011. Participants completed baseline surveys following randomization, and follow up surveys were conducted every 3 months. The surveys collected every 3 months were done via-phone. These surveys collected information on residential stability, service use and vocational activities. Participants were asked to report in person to provide subjective accounts of physical and mental health and substance use every 6 months. These surveys were conducted in an institution if the participant was institutionalized, at the Centre for Research on Inner City Health in Toronto, Ontario, or at the participants’ homes upon request. Physiological measures such as height, weight and waist circumference were also performed every six months using a measuring tape and digital scale. The trial concluded after all participants had completed their final interviews at the 24 month follow-up period. Data collection ceased in 2013.

### BMI and Waist Circumference Definition

Body weight was measured using a portable digital scale, and height was measured using a portable measuring stick. Weight and height were measured twice to the nearest 0.1 kg and 0.1cm, respectively, and the mean of the two measures was used in the analysis. We calculated body mass index (BMI) as weight in kilograms divided by height in meters squared (weight [kg] / height [m^2^]). BMI was dichotomized into two categories: overweight and obese (BMI > 24.9) and not overweight or obese (BMI < = 24.9) [26]. Similarly, waist circumference was also dichotomized into two categories: overweight and obese (waist circumference > = 102cm for men or 88cm for women) or not overweight or obese (waist circumference < 102cm for men or 88cm for women) [26].

### Potential Confounders

Baseline socio-demographic exposures included age, gender, ethnicity (ethnoracial or aboriginal status) and duration of homeless. Self-rated health exposures such as the EQ5D were selected as well as self-reported substance use as potential confounders. Specific items of the MCAS scale were used to investigate participants’ self-reported cooperation, medical compliance and substance use. The Mini International Neuropsychiatric Interview (MINI) was used at the screening interview to provide a current diagnosis of mental illness and/or substance or alcohol use or dependence or dependence disorders [27].

### Statistical Analysis

We first present the social and demographic characteristics at baseline, identifying those variables that differed between treatment and TAU groups. The sample was later examined for these differences among those who had BMI and Waist Circumference at both time points. Using the cut-offs previously listed, we examined the distribution of BMI and Waist Circumference change for those whose changed from or to being overweight or obese. To determine whether the BMI and Waist Circumference at 24 months after randomization was significantly different from its baseline counter-part, an Analysis of Covariance (ANCOVA) was performed to assess whether there was post-test difference in BMI, adjusting for participant’s BMI at baseline. Variables found to be significantly different between the treatment and TAU groups at baseline were entered into this model stratified by need level. To address issues surrounding multiplicity, a p-value of p<0.01 was considered as significant.

Missing data in our sample was caused by participant refusal and loss to follow-up. An additional 14 pregnant, transgendered, and transsexual participants were excluded from the analyses. In an effort to reduce the amount of missing data, height measurements were carried forward to other time points to calculate BMI if only height data was missing (N = 12). We also examined whether differences existed for participants with BMI and waist circumference data at both time points (see S1 File) and for those who had missing BMI and waist circumference data at one or both time points. All analyses were conducted using SAS 9.4.

## Results

At baseline, there were 486 (84.52%) participants with height and weight measurements, whereas at 24 months there were only 267 (46.34%) participants with this measurement (22 of whom did not have a baseline height or weight). Similarly, at baseline 476 (82.78%) participants provided a waist circumference measurement whereas at 24 months, 261 (45.39) participants provided this measurement. For inclusion in the ANCOVA models, participants needed data for BMI and Waist Circumference at both baseline and 24 months. Participants who were diagnosed with a psychotic disorder or substance abuse were found to be more likely to have these measures missing at both time points. We conducted analyses using data from the 245 (43.67%) participants with BMI measurements at both time points and the 239 (42.60%) participants with waist circumference measurements at both time points.

Table 1 describes the distribution of participants by each need level group for those who had BMI and waist circumference data at each time point. The average age of participants was approximately 40, with males being the majority of both need level groups. The proportion of participants who were classified as “ethnoracial” (not White) was higher among the moderate needs group (64%) than the high needs group (44%). Mean time homeless ranged from 54.29 to 86.96 months. Moderate food insecurity was present with mean food insecurity scores ranging from 4.36 to 4.62. High rates of substance and alcohol dependence were observed in both the moderate and high needs groups. All participants met the criteria for a mental illness, with major depressive episodes most common in the moderate needs group and psychotic disorders most common in the high needs group.

**Table 1:** Baseline Characteristics of Toronto At Home/Chez Soi Participants (N = 561) stratified by Need Level.

	Moderate Needs				High Needs				
Variable	INT (N = 201)	TAU (N = 166)	Total (N = 367)	P Value	INT (N = 96)		TAU (N = 98)	Total (N = 194)	P Value
**Age (Mean, SD)**	39.51 (11.64)	40.89 (12.42)	40.13 (12.00)	0.28	37.85 (10.99)		41.18 (11.73)	39.54 (11.46)	0.04
**Gender (n, %)**									
Female	63 (31.34)	53 (31.93)	116 (31.61)	0.90	32 (33.33)		19 (19.39)	51 (26.29)	0.03
Male	138 (68.66)	113 (68.07)	251 (68.39)		64 (66.67)		79 (80.61)	143 (73.71)	
**Ethnoracial (n, %)**									
Yes	134 (66.67)	98 (59.04)	232 (63.22)	0.13	46 (47.92)		53 (54.08)	99 (51.09)	0.39
No	67 (33.33)	68 (40.96)	135 (36.78)		50 (52.08)		45 (45.92)	95 (48.97)	
**Aboriginal (n, %)**									
Yes	10 (4.98)	7 (4.22)	17 (4.63)	0.73	7 (7.29)		3 (3.06)	10 (5.15)	0.18
No	191 (95.02)	159 (95.78)	350 (95.37)		89 (92.71)		95 (96.64)	184 (94.85)	
**Any Alcohol Use (n, %)**									
Yes	101 (50.25)	73 (43.98)	174 (47.41)	0.23	56 (58.33)		49 (50.52)	105 (54.40)	0.28
No	100 (49.75)	93 (56.02)	193 (52.59)		40 (41.67)		48 (49.48)	88 (45.60)	
**More than one substance per day (including alcohol) (n, %)**									
Yes	56 (27.86)	46 (27.88)	102 (27.87)	1.00	35 (36.46)		32 (32.65)	67 (34.54)	0.58
No	145 (72.14)	119 (72.12)	264 (72.13)		61 (63.54)		66 (67.35)	127 (65.46)	
**MINI Diagnoses (YES)**									
**Major Depressive Episode (n, %)**	91 (45.27)	76 (45.78)	167 (45.50)	0.92	17 (17.71)		18 (18.37)	35 (18.04)	0.91
**Manic or Hypomanic Episode (n, %)**	25 (12.44)	16 (9.64)	41 (11.17)	0.40	13 (13.54)		6 (6.12)	19 (9.79)	0.08
**PTSD (n, %)**	60 (29.85)	43 (25.90)	103 (28.07)	0.40	14 (14.58)		10 (10.20)	24 (12.37)	0.35
**Panic Disorder (n, %)**	36 (17.91)	31 (18.67)	67 (18.26)	0.85	6 (6.25)		3 (3.06)	9 (4.64)	0.29
**Mood Disorder with Psychotic Features (n, %)**	38 (18.91)	31 (18.67)	69 (18.80)	0.96	23 (23.96)		25 (25.51)	48 (24.74)	0.80
**Psychotic Disorder (n, %)**	54 (26.87)	43 (25.90)	97 (26.43)	0.84	55 (57.29)		60 (61.22)	115 (59.28)	0.58
**Alcohol Dependence (n, %)**	48 (23.88)	53 (31.93)	101 (27.52)	0.09	61 (32.29)		29 (29.59)	60 (30.93)	0.68
**Substance Dependence (n, %)**	72 (35.82)	63 (37.95)	135 (36.78)	0.67	43 (44.79)		31 (31.63)	74 (38.14)	0.06
**Alcohol Abuse (n, %)**	30 (14.93)	17 (10.24)	47 (12.81)	0.18	16 (16.67)		16 (16.33)	32 (16.49)	0.95
**Substance Abuse (n, %)**	19 (9.45)	13 (7.83)	32 (8.72)	0.58	11 (11.46)		8 (8.16)	19 (9.79)	0.44
**Total length of Homelessness Months (Mean, SD)**	54.29 (67.36)	56.86 (68.23)	55.45 (67.67)	0.72	67.13 (77.40)		86.96 (88.44)	77.15 (83.53)	0.11
**Overall Health Status (EQ5D) (1–100)**	60.32 (24.53)	61.31 (23.72)	60.75 (24.14)	0.71	64.67 (24.49)		63.60 (27.03)	64.12 (25.77)	0.78
**Overall Mental Health Status (EQ5D) (1–100)**	52.42 (25.98)	56.72 (25.76)	54.14 (25.91)	0.25	56.77 (28.32)		60.48 (31.64)	58.47 (29.80)	0.53
**Overall Physical Health Status (EQ5D) (1–100)**	58.78 (25.31)	66.58 (24.09)	61.92 (25.06)	0.03	62.82 (27.36)		63.08 (29.06)	62.94 (28.01)	0.96
**Food Securities Count (0–10)**	4.62 (2.57)	4.57 (2.49)	4.59 (2.53)	0.85	4.36 (2.51)		4.62 (2.55)	4.49 (2.52)	0.48
**Medication Compliance (MCAS) (1–5)**	3.99 (0.97)	4.19 (0.82)	4.08 (0.91)	0.03	3.00 (1.18)		3.26 (1.20)	3.13 (1.20)	0.14
**Cooperation with Treatment Providers (MCAS) (1–5)**	4.08 (0.61)	4.29 (0.63)	4.13 (0.62)	0.11	3.33 (0.92)		3.45 (0.81)	3.39 (0.87)	0.33
**Alcohol/drug Abuse (MCAS) (1–5)**	3.75 (1.25)	3.70 (1.32)	3.73 (1.28)	0.73	3.14 (1.43)		3.39 (1.38)	3.26 (1.41)	0.21
**Impulse Control (MCAS) (1–5)**	4.01 (0.81)	4.00 (0.87)	4.01 (0.84)	0.96	3.33 (0.95)		3.42 (0.94)	3.38 (0.94)	0.53
**BMI [kg] / [m** ^**2**^ **]**	26.03 (5.63)	26.63 (6.97)	26.30 (6.26)	0.39	26.87 (5.75)		26.16 (5.36)	26.49 (5.54)	0.43
**Waist Circumference (cm)**	90.86 (14.10)	93.32 (15.48)	91.94 (14.75)	0.14	96.27 (17.10)		94.09 (14.92)	95.14 (16.00)	0.40

For the high needs groups, significant differences were observed for age and gender (p = 0.04 and p = 0.02 respectively) with the intervention group having an approximate average age of 38 and the treatment as usual group with an age of 41. Similarly, the intervention group had a greater proportion of females (33%) compared to the treatment as usual (19%), whereas there was a greater proportion of males in treatment as usual group (81%) than the intervention (61%). For the moderate needs group, significant differences were observed for the overall physical health and the medication compliance item of the MCAS questionnaire. Participants in the intervention group reported a lower physical health score of 59 whereas those in the treatment as usual group had an average of 67. Tests of association were also performed for the sub-group of participants who had both measures at baseline and 24M for BMI and Waist Circumference to determine whether baseline differences were still present. The only difference was for the moderate needs group with the EQ5D Physical Health Component (p = 0.0367) among participants who had their Waist Circumference measured at both time points. Results of these comparisons are provided as supporting information.

### Obesity Characteristics

Categorical changes in BMI and waist circumference from baseline to 24 months are displayed in Table 2 for our analytic sample. Approximately 12 (5%) participants were overweight or obese at baseline but were not at 24 months, whereas 28 (11%) became overweight or obese at 24 months. The average change in BMI among participants who became obese was 4.43 ± 3.19 units with a maximum difference of ± 12.80 BMI units. The majority of participants (84%) experienced no change in their BMI categorizations over the 24 month trial period.

**Table 2:** Changes in Overweight/Obesity status from Baseline to 24 Months.

Overweight/Obese Status		BMI(N = 245)	Waist Circumference (N = 239)
Baseline	24 Months	N (%)	N (%)
**Not Overweight/Obese**	**Not Overweight/Obese**	96 (39.18)	137 (57.32)
**Obese**	**Obese**	109 (44.49)	60 (25.10)
**Not Overweight/Obese**	**Overweight/Obese**	28 (11.43)	28 (11.72)
**Overweight/Obese**	**Not Overweight/Obese**	12 (4.90)	14 (5.86)

BMI was dichotomized into two categories: obese and overweight (BMI > 24.9) and not obese or overweight (BMI < = 24.9). BMI was calculated as weight in kilograms divided by height in meters squared (weight (kg)/height (m2)).

Similar to the findings for BMI, the majority of participants (82.72%) experienced no categorical change in waist circumference. A higher proportion of participants (11.72%) went from not overweight or obese to overweight or obese than those who moved to the not overweight or obese category (5.56%). The waist circumference analysis shows a lower proportion of participants were classified as overweight or obese at some point in time (30.96%) as compared to BMI (49.39%). These results are displayed in Table 2

### Moderate Needs

In the moderate needs group, there was a non-significant treatment effect with an estimated difference between groups of 0.00063 (p = 0.99) for BMI (Table 3). Therefore, the Housing First with ICM intervention did not have an impact BMI. Both treatment groups experienced a small (β = 0.87, p < .001), yet significant, increase in BMI from baseline to 24 months.

**Table 3:** ANCOVA Results for Changes in Body Mass Index (BMI) and Waist Circumference from Baseline to 24 Months.

	BMI				Waist Circumference			
	Moderate Needs		High Needs		Moderate Needs		High Needs	
Independent Variables	Β	p-value	Β	p-value	β	p-value	β	p-value
**Housing First (ref: Treatment as Usual)**	0.00063	0.99	0.91	0.34	1.01	0.52	2.10	0.64
**BMI/Waist Circumference at Baseline** *	0.87	< .001	0.88	< .001	0.81	< .001	0.61	< .001

*For BMI model, BMI at Baseline was used as a predictor whereas for the Waist Circumference models the Waist circumference at baseline was used

Similarly for waist circumference, there was a non-significant treatment difference (β = 1.01, p = 0.52) and a small (β = 0.81, p<0.001), yet significant, increase in waist circumference from baseline to 24 months. Housing First with ICM had no significant impact on waist circumference.

### High Needs

For the high needs group, there was a non-significant treatment difference of 0.91 (p = 0.34) for BMI (Table 3) and similar to the moderate needs group, there was a small significant difference in the BMI over the study period (β = 0.88, p = <0.001). The Housing First with ACT intervention had no significant impact on BMI.

For the waist circumference, there was a non-significant treatment difference of 2.10(p = 0.64). Both the intervention and control groups experienced a small, yet significant, increase in waist circumference from baseline to 24 months (β = 0.61, p = < .001). Correspondingly, the Housing First with ACT intervention did not significant impact on waist circumference.

## Discussion

The primary objective of At Home/Chez Soi was to measure whether or not a Housing First intervention could be successful in stably housing participants while improving a variety of health and social outcomes [19] [12]. Therefore, additional data were collected at the Toronto study site to describe the basic health status of participants [20]. Analysis of the health status data on weight at the baseline and the 24 month points illustrated a high prevalence of overweight and obesity status, determined by BMI and waist circumference, in study participants at both time points.

As previously noted, home-based food preparation, the use of psychotropic drugs, and substance use all impact weight status [21] [22] [23] [24] [25]. We hypothesized that the intervention could have a positive or negative impact on BMI and waist circumference. However, we found no significant difference between those who had received stable housing and those who had not, indicating that receiving housing and supports through the Housing First intervention was not associated with significant changes in BMI or waist circumference.

Our findings confirms that extreme poverty, which persisted in both groups regardless of housing status [20], also contributes to unhealthy weights [28] [29]. Although the literature on homelessness and weight status is limited, there is a large amount of research that points to high rates of food insecurity and nutritional inadequacy in homeless and low-income populations [30] [31] [32]. Food insufficiency is one of the factors associated with poor health outcomes in the homeless population [5]. A study conducted in Toronto reveals that meal programs supplied by charitable organizations are nutritionally inadequate and do not supply adults with enough fruits, vegetables, or dairy products [33]. Decreased fruit and vegetable consumption contributes to overweight and obesity [34]. Hot meal programs (e.g. soup kitchens, meals provided at drop-in centres and shelters) are frequently accessed by individuals who experience chronic homelessness, severe poverty, low-income, and shelter use.

Toronto is a service rich environment [20]. In other words, regardless of housing status, participants are able to access a wide variety of programs which provide support for meeting basic needs. Although housed, participants who received the intervention continued to be low-income (Stergiopoulos et al., 2014 report). They may have continued to access hot meal programs and food banks, reserving their limited income to meet other basic needs and purchase other consumables. Future research should investigate what sources of food were accessed by this population both prior to and after receiving housing. In addition to food insecurity, persons living in poverty also have limited access to recreational programs that promote exercise and physical fitness [35]. Participation in physical exercise contributes to weight loss in overweight or obese persons [36]. Future work could analyze physical activity levels after receiving access to housing.

As this was one of the first studies to investigate the impact of Housing First on BMI and waist circumference, we were uncertain as to what the impact of the intervention would be on weight measures. The absence of a treatment effect indicates that providing homeless persons with stable housing and access to ICM and ACT treatments was not effective in reducing overweight and obesity in this population. This suggests the need to generate a better understanding of what resources (e.g. nutritional supports and recreational programing) are needed to assist Housing First participants with reaching and maintaining healthy weights. The persistence of high overweight and obesity rates in both the treatment and control groups suggests a need for policy and resource allocation to improve the quality of food provided to economically disadvantaged populations through hot meal programs and food banks. Low-income, homeless, and formerly homeless persons may benefit from an increased availability of low-barrier exercise and nutrition programs. Additionally, street outreach and shelter provider agencies may better assist clients in obtaining health weights by hiring staff nutritionists and recreation therapists. Increased general staff training on nutrition and exercise may also benefit clients.

Missing data for BMI and waist circumference and data attrition were limitations in this study. Fifty three participants had no BMI data at both baseline and 24 months and 263 participants were missing data at either baseline or 24 months. For waist circumference, 63 participants had no data at both baseline and 24 months and 259 had missing data at either baseline or 24 months. However, those with missing data were not substantially different from those for whom data were available. Additionally, significant differences were accounted for; therefore, the findings from our study retain generalizability. Participant attrition and non-response resulted in missing data. High attrition rates are common when conducting longitudinal research with vulnerable populations [37]. Future researchers should focus on recruiting and maintaining larger numbers of participants to increase the statistical power of the models. Additionally, future research is needed to determine the factors that influence participation in weight-related measures in populations experiencing extreme marginalization.

## Conclusion

The findings from this study indicate that Housing First interventions with both ICM and ACT supports have no significant impact on BMI and waist circumference. As this is the first study to investigate the impacts of Housing First on weight outcomes, additional research is needed to determine which supports are necessary and could be included to improve weight status in individuals who receive housing through Housing First interventions.

## Supporting Information

S1 CONSORT Checklist Consort 2010: Checklist of Information to Include when Reporting a Randomised Trial. (DOC)

S1 File Adjusted Baseline Comparisons for participants with complete data for BMI (N = 242) and Waist Circumference (N = 239) at Baseline and 24 Months. (DOCX)

S1 Protocol Study Protocol. (DOCX)
