# Supplementary material for: The Impact of a 24 Month Housing First Intervention on Participants’ Body Mass Index and Waist Circumference: Results from the At Home / Chez Soi Toronto Site Randomized Controlled Trial
Source: PLoS One. 2015 Sep 29;10(9):e0137069. doi: 10.1371/journal.pone.0137069 (PMC4587845; doi:10.1371/journal.pone.0137069)
Supplement: S1 File — (DOCX) [file pone.0137069.s002.docx]

**Adjusted Baseline Comparisons for participants with complete data for BMI (N=242) and Waist Circumference (N=239) at Baseline and 24 Months**

|  | BMI | | | | | | |  | Waist Circumference | | | | | | |
| --- | --- | --- | --- | --- | --- | --- | --- | --- | --- | --- | --- | --- | --- | --- | --- |
|  | Moderate Needs | | |  | High Needs | | |  | Moderate Needs | | |  | High Needs | | |
| Variable | INT (N=110) | TAU (N=68) | Total (N=178) |  | INT (N=36) | TAU (N=28) | Total (N=64) |  | INT (N=107) | TAU (N=66) | Total (N=173) |  | INT (N=37) | TAU (N=29) | Total (N=66) |
| **Age (Mean, SD)** | 40.37 (11.33) | 39.87 (11.25) | 40.18 (11.27) |  | 37.19 (10.34) | 40.50 (10.19) | 38.64 (10.32) |  | 40.36 (11.52) | 39.95 (11.13) | 40.21 (11.34) |  | 36.73 (10.52) | 39.97 (10.41) | 38.15 (10.51) |
| **Gender** |  |  |  |  |  |  |  |  |  |  |  |  |  |  |  |
| Female | 32 (29.09) | 17 (25.00) | 49 (27.53) |  | 11 (30.56) | 4 (14.29) | 15 (23.44) |  | 31 (28.97) | 17 (25.76) | 48 (27.75) |  | 11 (29.73) | 4 (13.79) | 15 (22.73) |
| Male | 78 (70.91) | 51 (75.00) | 129 (72.47) |  | 25 (69.44) | 24 (85.71) | 49 (76.56) |  | 76 (71.03) | 49 (74.24) | 125 (72.25) |  | 26 (70.27) | 25 (86.21) | 51 (77.27) |
| **Ethnoracial** |  |  |  |  |  |  |  |  |  |  |  |  |  |  |  |
| Yes | 75 (68.18) | 39 (57.35) | 114 (64.04) |  | 16 (44.44) | 12 (42.86) | 28 (43.75) |  | 73 (68.22) | 38 (57.58) | 111 (64.16) |  | 17 (45.95) | 13 (44.83) | 30 (45.45) |
| No | 35 (31.82) | 29 (42.65) | 64 (35.96) |  | 20 (55.56) | 16 (57.14) | 36 (56.25) |  | 34 (31.78) | 28 (42.42) | 62 (35.84) |  | 20 (54.05) | 16 (55.17) | 36 (54.55) |
| **Aboriginal** |  |  |  |  |  |  |  |  |  |  |  |  |  |  |  |
| Yes | 4 (4.98) | 3 (4.22) | 7 (4.63) |  | 2 (5.56) | 2 (7.14) | 4 (6.25) |  | 4 (3.74) | 3 (4.55) | 7 (4.05) |  | 2 (5.41) | 2 (6.90) | 4 (6.06) |
| No | 106 (96.36) | 65 (95.59) | 171 (95.37) |  | 34 (94.44) | 26 (92.86) | 60 (93.75) |  | 103 (96.26) | 63 (95.45) | 166 (95.95) |  | 35 (94.59) | 27 (93.10) | 62 (93.94) |
| **Any Alcohol Use** |  |  |  |  |  |  |  |  |  |  |  |  |  |  |  |
| Yes | 59 (53.64) | 25 (36.76) | 84 (47.19)***** |  | 22 (61.11) | 17 (60.71) | 39 (60.94) |  | 54 (50.57) | 25 (37.88) | 79 (45.66) |  | 23 (62.16) | 18 (62.07) | 41 (62.12) |
| No | 51 (46.36) | 43 (63.24) | 94 (52.81) |  | 14 (38.89) | 11 (39.29) | 25 (39.06) |  | 53 (49.53) | 41 (62.12) | 94 (54.34) |  | 14 (37.84) | 11 (37.93) | 25 (37.88) |
| **More than one substance per day (including alcohol)** |  |  |  |  |  |  |  |  |  |  |  |  |  |  |  |
| Yes | 31 (28.18) | 20 (29.41) | 51 (28.65) |  | 14 (38.89) | 14 (50.00) | 28 (43.75) |  | 26 (24.30) | 20 (30.30) | 46 (26.59) |  | 15 (40.54) | 15 (51.72) | 30 (45.45) |
| No | 79 (71.82) | 48 (70.59) | 127 (71.35) |  | 22 (61.11) | 14 (50.00) | 36 (56.25) |  | 81 (75.70) | 46 (69.70) | 127 (73.41) |  | 22 (59.46) | 14 (48.28) | 36 (54.55) |
| **MINI Diagnoses (YES)** |  |  |  |  |  |  |  |  |  |  |  |  |  |  |  |
| **Major Depressive Episode** | 50 (45.45) | 30 (44.12) | 80 (44.94) |  | 7 (19.44) | 8 (28.57) | 15 (23.44) |  | 49 (45.79) | 28 (42.42) | 77 (44.51) |  | 6 (16.22) | 9 (31.03) | 15 (22.73) |
| **Manic or Hypomanic Episode** | 14 (12.73) | 5 (7.35) | 19 (10.67) |  | 6 (16.67) | 4 (14.29) | 10 (15.63) |  | 13 (12.15) | 5 (7.58) | 18 (10.40) |  | 5 (13.51) | 4 (13.79) | 9 (13.64) |
| **PTSD** | 34 (30.91) | 18 (26.47) | 52 (29.21) |  | 6 (16.67) | 4 (14.29) | 10 (15.63) |  | 32 (29.91) | 18 (27.27) | 50 (28.90) |  | 6 (16.22) | 5 (17.24) | 11 (16.67) |
| **Panic Disorder** | 21 (19.09) | 13 (19.12) | 34 (19.10) |  | 3 (8.33) | 2 (7.14) | 5 (7.81) |  | 20 (18.69) | 14 (21.21) | 34 (19.65) |  | 3 (8.11) | 2 (6.90) | 5 (7.58) |
| **Mood Disorder with Psychotic Features** | 24 (21.82) | 11 (16.18) | 35 (19.66) |  | 8 (22.22) | 10 (35.71) | 18 (28.13) |  | 23 (21.50) | 11 (16.67) | 34 (19.65) |  | 7 (18.92) | 11 (37.93) | 18 (27.27) |
| **Psychotic Disorder** | 22 (20.00) | 15 (22.06) | 37 (20.79) |  | 22 (61.11) | 12 (42.86) | 34 (53.13) |  | 21 (19.63) | 15 (22.73) | 36 (20.81) |  | 23 (62.16) | 13 (44.83) | 36 (54.55) |
| **Alcohol Dependence** | 32 (29.09) | 26 (38.24) | 58 (32.58) |  | 12 (33.33) | 10 (35.71) | 22 (34.38) |  | 29 (27.10) | 25 (37.88) | 54 (31.21) |  | 12 (32.43) | 11 (37.93) | 23 (34.85) |
| **Substance Dependence** | 38 (34.55) | 32 (47.06) | 70 (39.33) |  | 16 (44.44) | 11 (39.29) | 27 (42.19) |  | 34 (31.78) | 31 (46.97) | 65 (37.57)***** |  | 16 (43.24) | 11 (37.93) | 27 (40.91) |
| **Alcohol Abuse** | 14 (12.73) | 5 (7.35) | 19 (10.67) |  | 7 (19.44) | 6 (21.43) | 13 (20.31) |  | 14 (13.08) | 5 (7.58) | 19 (10.98) |  | 7 (18.92) | 6 (20.69) | 13 (19.70) |
| **Substance Abuse** | 7 (6.36) | 2 (2.94) | 9 (5.06) |  | 8 (22.22) | 4 (14.29) | 12 (18.75) |  | 7 (6.54) | 2 (3.03) | 9 (5.20) |  | 9 (24.32) | 4 (13.79) | 13 (19.70) |
| **Total length of Homelessness Months (Mean, SD)** | 49.43 (57.29) | 62.68 (71.03) | 54.52 (63.06) |  | 76.03 (84.39) | 79.11 (81.26) | 77.40 (82.36) |  | 50.67 (59.28) | 61.66 (68.11) | 54.89 (62.85) |  | 76.14 (82.88) | 77.41 (80.32) | 76.71 (81.12) |
| **Overall Health Status (EQ5D)** | 61.50 (24.84) | 62.03 (23.69) | 61.70 (24.33) |  | 65.64 (58.19) | 67.11 (57.76) | 66.31 (22.30) |  | 61.08 (24.99) | 61.93 (24.07) | 61.41 (24.57) |  | 67.82 (21.66) | 66.17 (24.21) | 67.06 (22.69) |
| **Overall Mental Health Status (EQ5D)** | 49.30 (26.29) | 59.51 (23.53) | 52.47 (25.79) |  | 60.60 (24.09) | 52.00 (30.75) | 58.14 (25.99) |  | 48.41 (25.99) | 60.17 (23.65) | 52.01 (25.76)***** |  | 60.60 (24.09) | 52.00 (30.75) | 58.14 (25.99) |
| **Overall Physical Health Status (EQ5D)** | 60.96 (25.11) | 70.29 (23.16) | 63.85 (24.79) |  | 65.60 (24.01) | 59.20 (31.76) | 63.77 (26.13) |  | 60.38 (25.06) | 69.97 (23.49) | 63.32 (24.86) |  | 65.60 (24.01) | 59.20 (31.76) | 63.77 (26.13) |
| **Food Securities Count** | 4.77 (2.67) | 4.15 (2.27) | 4.53 (2.54) |  | 4.53 (2.48) | 4.89 (2.45) | 4.69 (2.46) |  | 4.71 (2.62) | 4.18 (2.30) | 4.51 (2.51) |  | 4.46 (2.48) | 5.00 (2.48) | 4.70 (2.47) |
| **Medication Compliance (MCAS)** | 4.02 (0.83) | 4.19 (0.89) | 4.08 (0.85) |  | 2.83 (1.18) | 3.25 (1.24) | 3.02 (1.21) |  | 4.03 (0.83) | 4.18 (0.89) | 4.09 (0.86) |  | 2.84 (1.17) | 3.28 (1.22) | 3.03 (1.20) |
| **Cooperation with Treatment Providers (MCAS)** | 4.05 (0.59) | 4.15 (0.58) | 4.09 (0.58) |  | 3.26 (0.95) | 3.46 (1.00) | 3.35 (0.97) |  | 4.07 (0.60) | 4.17 (0.60) | 4.10 (0.60) |  | 3.28 (0.94) | 3.48 (0.99) | 3.37 (0.96) |
| **Alcohol/drug Abuse (MCAS)** | 3.76 (1.21) | 3.63 (1.30) | 3.71 (1.24) |  | 3.19 (1.41) | 3.46 (1.23) | 3.31 (1.33) |  | 3.80 (1.21) | 3.59 (1.30) | 3.72 (1.25) |  | 3.16 (1.36) | 3.44 (1.21) | 3.29 (1.30) |
| **Impulse Control (MCAS)** | 4.04 (0.81) | 3.87 (0.93) | 3.97 (0.86) |  | 3.39 (0.96) | 3.43 (0.96) | 3.41 (0.95) |  | 4.06 (0.82) | 3.88) | 3.99 (0.87) |  | 3.40 (0.96) | 3.41 (0.95) | 3.41 (0.94) |
| ***- P < 0.05**  ****-P < 0.01** |  |  |  |  |  |  |  |  |  |  |  |  |  |  |  |

Units of measurement for all variables are identical to the units of measurement provided in Table 1
