# Supplementary material for: The Impact of a 24 Month Housing First Intervention on Participants’ Body Mass Index and Waist Circumference: Results from the At Home / Chez Soi Toronto Site Randomized Controlled Trial
Source: PLoS One. 2015 Sep 29;10(9):e0137069. doi: 10.1371/journal.pone.0137069 (PMC4587845; doi:10.1371/journal.pone.0137069)
Supplement: Supplementary file 3 [file pone.0137069.s003.docx]

Study Protocol

"At Home" Project
Research Demonstration Project in Mental Health & Homelessness: Main RCT Study

1. Background and Rationale

Homelessness is a significant social problem that affects thousands of people every night in cities across Canada. A study by the City of Toronto indicates that at least 5,052 individuals are absolutely homeless in Toronto on any given night [1].

The prevalence of mental health problems and addictions among homeless people is significantly higher than in the general population. The literature suggests that between one-fourth and one-third of the homeless have serious mental illnesses such a schizophrenia, major depressive disorder or bipolar affective disorder [2,3]. Research also suggests that homeless people in Toronto have complex, and often unmet, mental health needs [4,5].

Almost half of Toronto's 2.6 million residents are immigrants including a large proportion of refugee claimants in Canada. A 1999 report by the City of Toronto, identified immigrants and refugees as one of five groups that are particularly vulnerable and in need of targeted strategies [6]. However, the literature concerning the overlap between homelessness, mental illness and ethnicity, race or culture is scant, particularly in Canadian settings.

Overall, there have been only a small number of studies that have examined the effectiveness of housing and support interventions for people with mental illness who are homeless and all of these studies have been conducted in the United States. Research in this area reveals that programs that provide housing and support to people with severe mental illness are effective in reducing homelessness and hospitalizations and in producing other positive outcomes [7]. More data are needed that considers both the unique circumstances of ethno-racial groups and homeless people generally within the services and systems available in Canada.

2. Study Overview and Objectives

This goal of this study is to examine the effectiveness and cost effectiveness of providing housing and support services for homeless adults in Toronto who have serious mental health issues. Specifically, the study will examine the effectiveness of the Housing First model plus either (1) Assertive Community Treatment services or (2) Intensive Case Management services, compared with usual care. A particular focus of this study will be to examine the effectiveness of Housing First with Intensive Case Management services designed specifically for homeless adults from racialized ethno-racial groups. *For details about the models being examined see 3-I & 3-J below and Appendix L: Study Intervention and Usual Care Group Details.*

This research is part of a larger multi-site study sponsored by the Mental Health Commission of Canada. The Mental Health Commission of Canada is a federally funded, arms-length, non-profit organization with a mandate to focus national attention on mental health. Toronto is one of five cities (including Vancouver, Winnipeg, Montréal and Moncton) across Canada where research projects with a common core research design will take place. The Mental Health Commission of Canada's National Research Team manages the multi-site study and works
